# Coup-de-Soleil, or Sunstroke

**Published:** 1854-07

**Authors:** 


Coup-de-soleil, or Sunstroke.—In connection with the purpose of the foregoing article, we append a few general hints on a severe and not unfrequently a fatal casualty, which is apt to occur during the hot months of our summer, especially in a crowded city, and principally among those engaged in active and violent exertion while exposed to the rays of the sun, and hence called *coup-de-soleil*, or *sunstroke.*

Many fatal errors are committed in the first moments of alarm on the occurrence of this accident,—errors, the evil consequences of which all the subsequent skill and care of the physician are sometimes ineffectual in remedying. The prominent symptoms of the attack are giddiness, faintness, amounting in some instances to complete insensibility or apparent death.

The first and best thing to be accomplished, is to remove the patient into a cool room or shady spot, laying him carefully on the back, with the head very slightly, if at all, raised. *Send for a physician.* Sprinkle the face with cool water, untie all strings, handkerchiefs, or bandages from the throat, chest, or waist. Rub the hands and feet briskly, and if the patient can swallow, administer a little brandy and water, or a little spirits of hartshorn in water, *in small quantities at a time.* Promote free respiration by fanning and preventing the crowding of persons around. Prevent strenuously the drinking of large draughts of cold water, however urgent the request of the patient may be, until reaction takes place.

If there should be any delay in the arrival of a physician, and the patient should not rally, the rubbing of the limbs must be continued, and in addition, mustard poultices may be applied over the soles of the feet and the pit of the stomach, and an emetic of mustard and water may be administered, particularly if the patient, as is too often the case, immediately previous to his seizure, has been drinking cold water in large quantity. Such precautions judiciously, consistently, and perseveringly used, will frequently restore animation and produce reaction, after which the patient must be treated by a physician.—*Am. Medical Monthly.*

